# Global advances and future directions in lung cancer care: expert consensus and strategic priorities

**DOI:** 10.1016/j.esmoop.2025.106034

**Published:** 2026-01-23

**Authors:** M.-L. Meyer, S. Peters, P.A. Jänne, T.S. Mok, P.A. Bunn, N. Abdel Karim, N.K. Altorki, O. Arrieta, J. Bar, F. Cappuzzo, D. Carbone, S. Dacic, M. Diehn, R. Dziadziuszko, E. Felip, R. Flores, N. Florez, P.M. Forde, J.F. Gainor, J.E. Gray, L. Gros, B. Halmos, C.I. Henschke, R. Herbst, J.V. Heymach, K. Kelly, M.G. Kris, N.B. Leighl, C. Mathias, T.U. Marron, J.L. Mulshine, J. Naidoo, R. Osarogiagbon, A. Passaro, N. Peled, D. Planchard, K. Politi, S. Popat, B. Pyenson, K.L. Reckamp, S.H. Ren, G.J. Riely, C. Rolfo, C.M. Rudin, T. Sen, M.D. Shields, H. Singh, F. Skoulidis, B.J. Solomon, R. Stahel, B.M. Stiles, K. Syrigos, J.C.-H. Yang, P.-C. Yang, A. Wolf, A.J. Wozniak, S.N. Waqar, H. Wakelee, F.R. Hirsch

**Affiliations:** 1 Department of Oncology, CHUV—University of Lausanne, Lausanne, Switzerland; 2 Dana-Farber Cancer Institute and Harvard Medical School, Boston, USA; 3 State Key Laboratory of Translational Oncology, Department of Clinical Oncology, The Chinese University of Hong Kong, Hong Kong, China; 4 Division of Medical Oncology, Department of Medicine, University of Colorado School of Medicine, Aurora, USA; 5 George Washington University Cancer Center, Washington, USA; 6 Department of Thoracic Surgery, Weill Medical College of Cornell University, New York, USA; 7 Thoracic Oncology Unit, Instituto Nacional de Cancerología (INCan), Mexico City, Mexico; 8 Jusidman Cancer Center, Sheba Medical Center, Ramat Gan, Israel; 9 Sackler Faculty of Medicine, Tel Aviv University, Tel Aviv, Israel; 10 Regina Elena Institute for Cancer Research, Rome, Italy; 11 Ohio State University Comprehensive Cancer Center—Arthur G. James Cancer Hospital and Richard J. Solove Research Institute, Columbus, USA; 12 Department of Pathology, Yale School of Medicine, New Haven, USA; 13 Department of Radiation Oncology, Stanford University School of Medicine/Stanford Cancer Institute, Stanford, USA; 14 Department of Oncology and Radiotherapy and Early Phase Clinical Trials Centre, Medical University of Gdańsk, Gdańsk, Poland; 15 Vall d’Hebron University Hospital and Vall d’Hebron Institute of Oncology (VHIO), Barcelona, Spain; 16 Department of Thoracic Surgery, Icahn School of Medicine at Mount Sinai, New York, USA; 17 Trinity St. James’s Cancer Institute, Trinity College Dublin, Dublin, Ireland; 18 H. Lee Moffitt Cancer Center and Research Institute, Tampa, USA; 19 Montefiore Medical Center and Albert Einstein College of Medicine, Bronx, USA; 20 Mount Sinai Hospital, New York, USA; 21 Department of Thoracic/Head and Neck Medical Oncology, MD Anderson Cancer Center, Houston, USA; 22 International Association for the Study of Lung Cancer (IASLC), Denver, USA; 23 Memorial Sloan Kettering Cancer Center and Weill Cornell Medical College, New York, USA; 24 Cancer Clinical Research Unit, Princess Margaret Cancer Centre, University Health Network, Toronto, Canada; 25 Oncoclínicas Bahia and Hospital Santa Izabel, Salvador, Brazil; 26 Center for Thoracic Oncology, Tisch Cancer Center, Icahn School of Medicine at Mount Sinai, New York, USA; 27 Center for Healthy Aging, Rush University Medical College, Chicago, USA; 28 Multidisciplinary Thoracic Oncology Program, Baptist Cancer Center, Memphis, USA; 29 European Institute of Oncology IRCCS, Milan, Italy; 30 Shaare Zedek Medical Center, Jerusalem, Israel; 31 Department of Cancer Medicine, Gustave Roussy, Villejuif, France; 32 Royal Marsden Hospital NHS Foundation Trust, London, UK; 33 Pyenson Healthcare Analytics, LLC, Madison, USA; 34 Cedars-Sinai Medical Center, Los Angeles, USA; 35 Department of Medical Oncology, Shanghai Pulmonary Hospital, Tongji University School of Medicine, Shanghai, China; 36 Division of Hematology/Oncology (Thoracic Oncology), Indiana University Melvin and Bren Simon Comprehensive Cancer Center, Indianapolis, USA; 37 Precision for Medicine, Washington, USA; 38 Department of Medical Oncology, Peter MacCallum Cancer Centre and University of Melbourne, Melbourne, Australia; 39 ETOP IBCSG Partners Foundation, Bern, Switzerland; 40 Oncology Unit, Department of Medicine, University of Athens, Athens, Greece; 41 National Taiwan University Cancer Center, Taipei, Taiwan; 42 Department of Internal Medicine, National Taiwan University Hospital and College of Medicine, Taipei, Taiwan; 43 Department of Thoracic Surgery, Icahn School of Medicine at Mount Sinai, One Gustave L. Levy Place, New York, USA; 44 Lung Cancer Research Foundation, New York, USA; 45 Division of Oncology, Department of Medicine, Washington University School of Medicine in St. Louis, St. Louis, USA

**Keywords:** lung neoplasm, early detection, immunotherapy, clinical trial, biomarker

## Abstract

**Background:**

Over the past decade, lung cancer management has been reshaped by advances in early detection, treatment, and prevention. Prevention now extends beyond tobacco control to include recognition of non-tobacco risk factors, screening, and incidental nodule programs. Yet progress in primary prevention remains uneven, with marked regional disparities. Smoking prevalence continues to decline and measures to reduce particulate matter exposure are expanding, but the overall global impact remains inconsistent.

**Patients and methods:**

This article draws upon the discussions and expert recommendations presented at the New York Lung Cancer Foundation Summit 2025, integrating perspectives on prevention, screening, therapeutic innovation, and health system challenges across diverse health care settings.

**Results:**

Screening programs, now active in >40 countries, achieve lower false-positive rates and earlier-stage diagnoses, although lung cancer incidence is rising among individuals who never used any tobacco products in some regions. Therapeutic innovations—including perioperative immunotherapy, targeted treatments for oncogene-driven non-small-cell lung cancer, and antibody–drug conjugates (ADCs)—have markedly improved survival outcomes. Persistent challenges include refining patient selection, sequencing multimodal therapies, managing toxicity, and understanding mechanisms of resistance. Systemic barriers such as unequal progress in tobacco and vaping prevention, limited screening uptake, delayed molecular testing, and restricted access to multidisciplinary care continue to blunt these gains. Ongoing research on novel immunotherapies, ADCs, and bispecific antibodies aims to overcome therapeutic resistance. In small-cell lung cancer, consolidation immunotherapy and delta-like ligand 3-targeted approaches have improved outcomes and are redefining treatment paradigms. Persistent disparities in access and trial participation underscore the need for more equitable study designs, stronger international collaboration, and clearer communication with the public.

**Conclusions:**

This article summarizes current advances and strategic priorities in lung cancer research and care, reflecting the discussions and recommendations of the New York Lung Cancer Foundation Summit 2025.

## Highlights

• Lung cancer remains a major global health burden.
• Screening adoption is expanding worldwide, enabling earlier-stage detection and improved outcomes.
• Therapeutic advances include perioperative immunotherapy, targeted therapies, ADCs, and bispecific antibodies.
• Biomarkers such as ctDNA, MRD, and molecular profiling are being investigated to guide treatment decisions.
• Global disparities, limited trial access, and implementation challenges continue to hinder progress.

## Introduction

**Figure 1:**
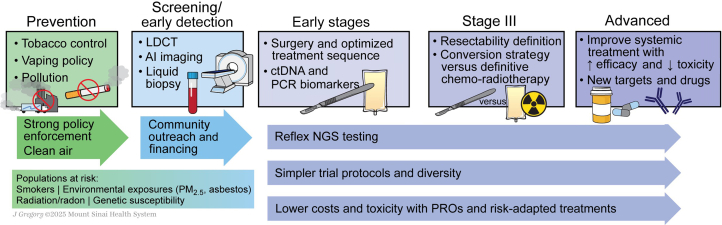
**Calls to action from the NYLCF Summit.** AI, artificial intelligence; ctDNA, circulating tumor DNA; LDCT, low-dose CT; NGS, next-generation sequencing; NYLCF, New York Lung Cancer Foundation; PROs, patient-reported outcomes.

Lung cancer remains the leading cause of cancer mortality worldwide.^1^ In the United States, the 3-year relative survival for individuals with non-small-cell lung cancer (NSCLC) improved from 26% in 2004 to 43% in 2018, with only improvement from 9% to 12% for individuals with small-cell lung cancer (SCLC).^2^ While progress in prevention, early detection, and therapy has been substantial, disparities in access and treatment resistance continue to hinder optimal outcomes.^3^ To address these challenges, the New York Lung Cancer Foundation (NYLCF) convened a multidisciplinary group to define priorities in research and care (Table 1). This article synthesizes the NYLCF 2025 Summit’s discussions into practical, stage-specific actions and system-level recommendations (Figure 1).

**Table 1:** Key calls to action

Section	Key calls to action
Prevention and early detection	Strengthen tobacco/vaping regulation
	Implement WHO FCTC
	Integrate lung screening into broader preventive care and manage implementation
	Guarantee access for underserved populations
Early-stage NSCLC	Ensure comprehensive biomarker testing
	Optimize perioperative ICI–CT sequencing
	Refine strategy in borderline resectable disease, evaluating role of neoadjuvant versus perioperative versus adjuvant therapy
	Study role of adjuvant therapy related to pCR and MPR after neoadjuvant therapy
	Accelerate umbrella trials for rare alterations
	Study ctDNA as predictive biomarker and standardize MRD-guided therapy escalation/de-escalation
	Systematically report QoL
	Clarify duration of targeted adjuvant therapy
Stage III NSCLC	Harmonize resectability definitions
	Study conversion strategies
	Refine consolidation therapy per driver status
	Optimize induction versus CRT sequencing
Advanced NSCLC without drivers	Personalize ICI versus CT–ICI
	Investigate new and composite biomarkers
	Develop strategies for ICI resistance
	Evaluate bispecifics and ADCs
	Explore new targets
Advanced NSCLC with drivers	Expand access to targeted therapy
	Study new combinations
	Establish sequencing algorithms
	Integrate re-biopsy and molecular resistance work-ups
	Support global trial participation for rare drivers
Small-cell lung cancer (SCLC)	Implement consolidation ICI in LS-SCLC integrate DLL3-targeted agents into ES-SCLC care
	Clarify PCI versus MRI surveillance
	Accelerate early-line trial enrollment
Diagnostics and biomarkers	Universalize reflex NGS
	Harmonize biomarker assays globally
	Invest in AI histology/radiomics
Collaborations and barriers	Foster pharma–academic–NGO partnerships
	Simplify regulatory frameworks; expand community-based trial infrastructure
	Establish pooled precompetitive funding
	Combat tobacco lobbying
Trial design and disparities	Simplify eligibility/exclusions criteria
	Adopt adaptive and pragmatic designs
	Incentivize diversity plans; decentralize assessments
	Integrate supportive measures (e.g. smoking cessation)
Equity and global inclusion	Expand trials in LMICs
	Build local sequencing hubs
	Align disparity reduction with stakeholder interests
	Promote local leadership
Conclusion/outlook	Sustain NIH/NCI/WHO/EC funding
	Maintain annual summit dialogues
	Create dedicated taskforces to track progress
	Expand IASLC/global collaborations
	Engage younger investigators

ADC, antibody–drug conjugate; AI, artificial intelligence; CRT, chemoradiotherapy; CT, chemotherapy; ctDNA, circulating tumor DNA; DLL3, delta-like ligand 3; EC, European Commission; ES-SCLC, extensive-stage small-cell lung cancer; FCTC, Framework Convention on Tobacco Control; IASLC, International Association for the Study of Lung Cancer; ICI, immune checkpoint inhibitor; LMICs, low- and middle-income countries; LS-SCLC, limited-stage small-cell lung cancer; MPR, major pathological response; MRD, minimal residual disease; MRI, magnetic resonance imaging; NCI, National Cancer Institute; NGO, non-governmental organization; NGS, next-generation sequencing; NIH, National Institutes of Health; NSCLC, non-small-cell lung cancer; PCI, prophylactic cranial irradiation; pCR, pathological complete response; QoL, quality of life; SCLC, small-cell lung cancer; TCE, T-cell engager; WHO, World Health Organization.

## Prevention and early detection

Global smoking prevalence and tobacco-attributable lung cancer mortality are declining, yet the absolute burden remains high—especially in low- and middle-income countries (LMICs).^4^ Effective measures include taxation, mass media campaigns, pictorial warnings, and smoke-free policies, with full implementation of the World Health Organization (WHO) Framework Convention on Tobacco Control.^4^^,^^5^

Low-dose CT screening is now active in >40 countries mostly based on smoking history, with low false-positive rates (<5% in mature programs),^6^, ^7^, ^8^, ^9^ enabling stage shift toward resectable disease.^10^ However, tobacco is not the sole risk factor; individuals exposed to environmental pollutants (PM_2.5_ or asbestos), those exposed to radiation or radon, and people with inherited susceptibility are also at increased risk.^11^ Indeed, lung cancer incidence in never smokers is rising—particularly in Asia—prompting the evaluation of risk models that incorporate family history and environmental exposures.^12^, ^13^, ^14^ As risk groups differ in absolute risk and disease trajectories, limiting the impact of uniform prevention and early detection strategies, tailored approaches should be further explored. Ongoing studies are evaluating the cost-effectiveness of biannual screening in defined lower-risk groups as well as evaluating the performance of relevant artificial intelligence (AI) tools and increasing screening accrual by co-recruiting with other programs (e.g. breast screening). Financing the screening encounter, covering downstream clinical management costs, and building community trust remain key for uptake.^5^^,^^14^, ^15^, ^16^ Research is also exploring areas such as liquid biopsy and fragmentomics.^17^^,^^18^

## Early-Stage NSCLC

### Early-stage NSCLC without molecular drivers

Surgery remains the standard of care for stage I NSCLC, while definitive radiotherapy is recommended for non-operable patients.^19^ For resectable stage II-III disease, neoadjuvant or perioperative strategies combining immune checkpoint inhibitors (ICIs) with chemotherapy are now preferred, having demonstrated gains in overall survival (OS) and event-free survival (EFS) with nivolumab, pembrolizumab, tislelizumab, and toripalimab.^20^, ^21^, ^22^, ^23^ Perioperative durvalumab has also shown an EFS benefit.^24^

Several questions remain, particularly regarding the definition of resectability and the role of post-operative ICI when used as part of a perioperative approach.^25^ Ongoing studies such as ADOPT-LUNG and PROSPECT-Lung (NCT06284317, NCT06632327) are evaluating the added value of adjuvant ICI. Pathological complete response (pCR) and circulating tumor DNA (ctDNA) are established prognostic biomarkers, and their utility as predictive markers is being investigated (NCT04585490, NCT04585477). The International Association for the Study of Lung Cancer (IASLC) is also assessing pCR as a potential regulatory endpoint.^26^ Across trials, pCR rates of ∼20%^20^^,^^21^^,^^24^^,^^27^^,^^28^ prompt discussions about expanding surgical eligibility—for instance, to borderline resectable stage III disease.^29^^,^^30^ However, ∼20% of patients enrolled in neoadjuvant or perioperative trials never proceed to surgery despite being considered resectable at baseline.^20^^,^^21^^,^^24^^,^^27^ Upfront surgery followed by adjuvant therapy remains a valid option in selected patients,^31^^,^^32^ although outcomes appear less favorable than with neoadjuvant approaches. Adjuvant concurrent chemo-immunotherapy is also under investigation (NCT04267848, NCT04564157).

Predictive biomarkers of response to ICI could help refine patient selection. In a cohort of 112 patients receiving neoadjuvant therapy, pCR was rare in never smokers and absent in those with *Kirsten rat sarcoma viral oncogene homolog* (*KRAS*)*/Serine/threonine kinase 11* (*STK11*) or *KRAS/Kelch-like ECH-associated protein 1* (*KEAP1*) co-mutation,^29^ suggesting that some molecular subgroups may benefit from dual ICI strategies, a concept warranting further study.^33^ An inflammatory score has been linked to overall response rate (ORR) and improved EFS with dual ICI, but prospective validation is needed.^33^ A direct comparison of dual ICI versus ICI–chemotherapy would be valuable.

The phase II NeoCOAST-2 trial (NCT05061550) is testing neoadjuvant combinations in resectable NSCLC, adding novel agents (oleclumab, monalizumab, volrustomig) to durvalumab, antibody–drug conjugates (ADCs), and chemotherapy. The datopotamab deruxtecan (Dato-DXd) arm achieved the highest pCR rate (35%) and a major pathological response (MPR) rate of 63%.^34^ Novel adjuvant strategies, including vaccines (NCT06312137, NCT06623422, NCT06077760) and ADCs (NCT06431633, NCT06564844), are under investigation. Proteomics and metabolomics could help predict benefit and toxicity.^35^

### Early-stage NSCLC with molecular drivers

The growing use of adjuvant therapies highlights the need for biomarker testing, even in early-stage disease.^36^ Adjuvant targeted treatment is now approved for *EGFR* or *ALK* alterations.^37^ In the ADAURA trial, 3 years of adjuvant osimertinib improved both disease-free survival (DFS) and OS in patients with stage IB-IIIA NSCLC [TNM tumor–node–metastasis) seventh edition].^38^ The ongoing ADAURA2 trial (NCT05120349) is evaluating osimertinib in earlier stages. For *ALK*-rearranged NSCLC, 2 years of adjuvant alectinib improved DFS.^39^ Adjuvant selpercatinib for rearranged during transfection-fusion-positive NSCLC is also under investigation (NCT04819100).

The role of neoadjuvant and perioperative targeted therapy is under investigation. In the phase II NEOS trial (stage IIA-IIIB, TNM 8th), neoadjuvant osimertinib achieved a 71% ORR, with 75% proceeding to surgery.^40^ In the phase III NeoADAURA trial (stage II-IIIB, TNM eight edition), osimertinib plus chemotherapy achieved a 26% MPR, though EFS data are immature.^41^ For potentially resectable stage III *ALK*-rearranged NSCLC, neoadjuvant alectinib induced ORR of 63% and 67% in two phase II trials.^42^^,^^43^ Additional studies are testing entrectinib and vemurafenib (NCT04302025, NCT04302025).

Phase III trials will be difficult to conduct for rarer alterations. For molecular subsets with proven efficacy in advanced disease (e.g. *ROS1, BRAF, EGFR* exon 20 insertions), panelists agreed that (neo)adjuvant use may be justified.

Biomarker-guided treatment adaptation, including minimal residual disease (MRD)-based escalation or de-escalation, is under active study in oncogene-addicted NSCLC.^44^ Research efforts, such as the LEADER trial (NCT04712877), aim to deepen understanding of tumor biology based on tumor and blood genomic testing.^45^ AI prediction from hematoxylin and eosin could also help.^46^ Intensification strategies should be tested in controlled trials to clarify how ctDNA/MRD can guide therapy. The panel emphasized the need to standardize stratification factors and outcome definitions (e.g. pCR, MRD negativity) and to predefine their integration into staging and trial design.

## Stage III NSCLC

Stage III NSCLC management depends on tumor resectability. Unresectable disease is typically treated with chemoradiotherapy followed by durvalumab.^19^^,^^37^ This paradigm is being challenged by neoadjuvant approaches and broader resectability criteria.^25^ Early data indicate that conversion surgery may be feasible in up to 70%-80% of selected initially unresectable patients.^47^^,^^48^ Adaptive strategies for marginally resectable disease are also under study (NCT05925530). Research focusing on optimizing immunotherapy have so far been negative,^49^, ^50^, ^51^, ^52^ though phase III trial is still ongoing (NCT05221840).

In *EGFR*-mutated tumors, *post hoc* analyses of the PACIFIC trial suggest limited benefit of ICI consolidation,^53^ while phase III trials of consolidation with osimertinib or aumolertinib have demonstrated improved EFS,^54^^,^^55^ establishing consolidation epidermal growth factor receptor (EGFR) tyrosine kinase inhibitor (TKI) as a new standard of care.^37^^,^^56^ The duration and strategies for other oncogenic alterations remain under investigation.

## Advanced-Stage NSCLC

### Advanced NSCLC without actionable alteration

The standard first-line treatment for advanced NSCLC without actionable alterations includes immunotherapy alone or combined with chemotherapy.^37^^,^^57^ For tumors with high programmed death-ligand 1 (PD-L1) expression (≥50%), anti-programmed cell death protein 1 (PD-1)/PD-L1 monotherapy is established, while the value of chemo-immunotherapy and sequencing is under study (NCT04547504, NCT03793179). Dual checkpoint blockade with anti-cytotoxic T-lymphocyte antigen-4 (CTLA-4) plus anti-PD-1, without chemotherapy, is an option for PD-L1 ≥1%, as per initial trial designs.^37^^,^^57^ In PD-L1-negative disease, chemo-immunotherapy remains standard, with dual ICI plus chemotherapy (e.g. CheckMate 9LA, POSEIDON) providing consistent OS benefit.^58^^,^^59^

*STK11* and *KEAP1* mutations are associated with poor prognosis and limited response to PD-(L)1 monotherapy,^60^ with signals of added activity from dual ICI.^60^^,^^61^ The TRITON phase IIIb trial is evaluating dual ICIs plus chemotherapy in this subgroup (NCT06008093).

For frail or elderly patients ineligible for platinum chemotherapy (ECOG PS 3 or age >80 years), anti-PD-(L)1 monotherapy is an option, regardless of PD-L1 status.^3^^,^^62^^,^^63^

New immunotherapies are under investigation, including novel CTLA-4-targeting antibodies such as gotistobart (NCT05671510) and botensilimab (NCT06322108). T cell immunoreceptor with Ig and ITIM domains-targeting agents have not demonstrated meaningful OS improvement in phase III trials.^64^^,^^65^

For lymphocyte activation gene 3 targeting antibodies, relatlimab plus nivolumab and chemotherapy has shown benefit in PD-L1-positive subgroups in a phase II trial,^66^ and the phase III RELATIVITY-1093 is ongoing (NCT06561386). Two phase III trials of cemiplimab plus fianlimab, with or without chemotherapy, are also under way (NCT05800015, NCT05785767). Eftilagimod alpha, a soluble LAG-3 protein that activates antigen-presenting cells,^67^ showed efficacy in PD-L1-high NSCLC in a phase II study. Several other LAG-3 agents are in development.^68^ Additional studied targets include T cell immunoglobulin and mucin domain-containing protein 3, natural killer cell protein group 2-A, cluster of differentiation 73, chemokine receptor 8, V-domain Ig suppressor of T cell activation, B and T lymphocyte attenuator, PVR related immunoglobulin domain containing, and B7 homolog 3.^69^ Resistance to immunotherapy remains poorly understood and can arise from both tumor-intrinsic factors and host immune dynamics.^70^^,^^71^

Targeting the vascular endothelial growth factor (VEGF) pathway has been evaluated across multiple therapeutic strategies. The IMpower150 trial demonstrated improved outcomes with the combination of atezolizumab, carboplatin, paclitaxel, and bevacizumab.^72^ Conversely, several anti-angiogenic or multitarget inhibitors have not improved survival when combined with immunotherapy (CONTACT-01, SAPPHIRE, LEAP-008, PRAGMATICA-Lung).^3^^,^^73^

A new generation of bispecific antibodies has emerged, aiming to concurrently inhibit VEGF and immune checkpoints. Ivonescimab, a dual VEGF/PD-1 antibody, improved progression-free survival (PFS) compared with pembrolizumab in a phase III trial in China among patients with PD-L1 ≥1% NSCLC.^74^ In HARMONi-6, ivonescimab plus chemotherapy achieved superior PFS versus tislelizumab plus chemotherapy in squamous NSCLC, with OS results pending.^75^ Additional VEGF/PD-1 bispecifics, including LM-299 (NCT06650566) and SSGJ-707 (NCT06361927), as well as the PD-L1/VEGF antibody BNT327 (NCT06712316, NCT06712355), are in development. Confirmation of survival benefit and broader global validation will be essential before this approach can be established.

Other bispecifics under investigation include volrustomig, targeting CTLA-4 and PD-1 (NCT05984277), rilvegostomig (PD-1/T cell immunoreceptor with Ig and ITIM domains), sabestomig (PD-1/T cell immunoglobulin and mucin domain-containing protein 3), and acasunlimab (4-1BB/PD-L1).^76^

ADCs also remain under investigation. Although the premise of delivering cytotoxic payloads via tumor-associated cell-surface targets is appealing, clinical efficacy has often shown poor correlation with target expression—with the exception of trastuzumab deruxtecan (T-DXd).^77^ Biomarker refinement is a key focus. For instance, Dato-DXd failed to improve OS in patients with advanced NSCLC,^78^ but showed higher ORR in patients with *EGFR*-mutated NSCLC, leading to a recent Food and Drug Administration (FDA) accelerated approval.^79^ Emerging strategies also explore metrics such as the trophoblast cell surface antigen 2 (TROP2)-normalized membrane ratio via quantitative scoring.^80^ Unfortunately, several ADCs have failed to improve outcome. Sacituzumab govitecan, targeting TROP2, failed to improve outcomes in the EVOKE-01 trial.^81^ Similarly, targeting CEACAM5 with tusamitamab ravtansine did not meet its dual primary endpoints of PFS and OS in the CARMEN-LC03 trial.^82^ Following negative results from the phase III HERTHENA-Lung02 trial, the Biologics License Application (BLA) for patritumab deruxtecan (HER3-DXd) was withdrawn.^83^ Combination strategies involving ADCs, ICIs, and chemotherapy are ongoing with promising results reported,^84^ and the key will likely lie in its toxicity profile and patient selection.

### Advanced NSCLC with actionable gene alteration

#### EGFR

Third-generation EGFR TKIs (osimertinib, aumolertinib, lazertinib, furmonertinib) are established first-line standards after demonstrating superiority over first-generation EGFR TKIs.^85^^,^^86^ Recent trials have expanded the treatment landscape, with two combinations improving both OS and PFS: chemotherapy plus osimertinib in FLAURA2,^87^ and amivantamab plus lazertinib in MARIPOSA.^88^ The MARIPOSA regimen requires prolonged amivantamab infusions with frequent infusion reactions^88^; but a subcutaneous formulation has shown reduced toxicity and non-inferiority.^89^ These advances raise questions about optimal regimen selection and sequencing. Central nervous system involvement, disease burden, toxicity profile, and access will have to be considered in treatment selection.

In *EGFR* exon 20 insertion-positive NSCLC, amivantamab + chemotherapy is the first-line standard.^90^ Next-generation TKIs are evaluated, with three phase III trials ongoing in first-line furmonertinib (NCT05607550), sunvozertinib (NCT05668988), and zipalertinib (NCT05973773).

Targeting VEGF is also promising. Combination of osimertinib and ramucirumab demonstrated improved PFS versus osimertinib alone in the RAMOSE trial.^91^ After progression on EGFR TKIs, ivonescimab demonstrated improved PFS combined with chemotherapy over chemotherapy, but without OS benefit.^92^ BNT307, which targets PD-L1 and VEGF-A, is also being studied in this population in combination with chemotherapy, with a reported ORR of 55% in a phase II trial.^93^

Resistance mechanisms pose increasing challenges.^86^ In the post-TKI setting, TROP2-directed ADCs have emerged as a key therapeutic option. Dato-DXd achieved a 45% ORR after EGFR TKI progression, leading to FDA accelerated approval.^79^ Sacituzumab tirumotecan demonstrated improvements in both PFS and OS compared with chemotherapy,^94^ supporting the global phase III TroFUSE-004 trial (NCT06074588). The drug is already approved in China.^95^

ADCs targeting HER3 (e.g. BL-B01D1) have also shown activity in early-phase trials.^86^ However, despite an initial PFS benefit, the BLA for HER3-DXd was voluntarily withdrawn due to negative OS results.^96^ For patients with *EGFR-*mutated NSCLC with MET amplification as a resistance mechanism, dual EGFR–MET inhibition is under investigation. The combination of osimertinib and tepotinib has shown an ORR of 50%,^97^ while the Chinese phase III SACHI trial reported an improved PFS with the combination of savolitinib and osimertinib versus chemotherapy.^98^ A global phase III trial (SAFFRON, NCT05261399) is ongoing. Combining EGFR TKIs with TKIs targeting BRAF, rearranged during transfection, or ALK also represents a promising strategy when these alterations emerge as resistance mechanisms.^86^

#### Other alterations

In *ALK*-rearranged NSCLC, the CROWN trial of first-line lorlatinib reported a median PFS of >5 years,^99^ solidifying its position as an excellent first-line treatment despite its challenging toxicity profile.^100^ Neladalkib, a fourth-generation ALK inhibitor, has demonstrated activity after lorlatinib failure,^101^ and is being evaluated head-to-head against alectinib in the first-line setting (ALKAZAR trial, NCT0676510). Concerns about long-term toxicities and fertility issues remain unresolved in this patient population.^102^ For patients with ROS1-positive advanced NSCLC, taletrectinib received FDA approval,^103^ based on the phase II TRUST trials, which demonstrated ORRs of 89% in TKI-naïve patients, and 55% in those previously treated with a ROS1 TKI.^104^ Zidesamtinib, another ROS1 TKI, reported ORR of 89% in treatment-naïve patients, and of 51% in patients with prior TKI.^105^ Other approved ROS1 inhibitors include entrectinib and repotrectinib,^106^^,^^107^ and the optimal treatment sequence remains an area of debate.

For human epidermal growth factor receptor 2 (*HER2*)-mutated NSCLC, T-DXd is approved in the post-first-line setting, with its first-line role under investigation (NCT05048797). T-DXd may also benefit tumors with high *HER2* expression (immunohistochemistry 3+), though with lower efficacy.^37^ Several new *HER2* inhibitors, including zongertinib and sevabertinib (BAY 2927088), are in development.^108^, ^109^, ^110^ Zongertinib showed durable responses and good tolerability,^111^ supporting the ongoing phase III BEAMion LUNG-2 trial (NCT06151574). The phase III SOHO-2 trial (NCT06452277) is comparing sevabertinib with chemo-immunotherapy. Another HER2-targeting ADC, SHR-A1811 (trastuzumab rezetecan), has demonstrated promising activity in pretreated patients and is advancing to phase II/III.^112^

*KRAS* is the most frequent driver mutation in Caucasian patients with NSCLC,^3^ and tumors harboring *KRAS* mutations typically respond to chemo-immunotherapy.^37^ To date, KRAS G12C-targeted therapies (sotorasib and adagrasib) have been reserved for second-line use.^37^New compounds also hold promise: divarasib demonstrated a 53% ORR^113^ and a phase III trial (Krascendo-1) is comparing divarasib with sotorasib and adagrasib in the second-line setting (NCT06497556). D3S-001 represents another inhibitor with improved target coverage with promising preliminary antitumor efficacy.^114^

Efforts are also under way to introduce KRAS G12C inhibitors into earlier lines of therapy. Recently, the KRYSTAL-7 trial reported an ORR of 59% and manageable safety with the combination of adagrasib and pembrolizumab in NSCLC harboring *KRAS* G12C mutations and PD-L1 ≥50%.^115^ Multiple other combinations of ICIs with KRAS inhibitors are also in progress, including olomorasib with pembrolizumab in the SUNRAY-01 trial (NCT06119581), divarasib with pembrolizumab (NCT06793215), MK-1084 with pembrolizumab (NCT06345729), and elironrasib (RMC-6291) with pembrolizumab, with or without chemotherapy, in the RMC-Lung-101 trial (NCT06128551).

Key challenges in targeting KRAS include a high rate of primary resistance and rapid molecular adaptation.^116^, ^117^, ^118^, ^119^ KRAS operates in two states: inactive (GDP-bound) and active (GTP-bound). The first-generation inhibitors, sotorasib and adagrasib, bind the inactive state, classifying them as ‘off-state’ inhibitors.^120^ Newer efforts aim to develop inhibitors targeting the active state for potentially improved efficacy,^121^ such as RMC-6291 (elironrasib) RMC-6236 (daraxonrasib)^122^^,^^123^ and the dual state inhibitors BBO-8520 (NCT06343402) and FMC-376 (NCT06244771).

Novel inhibitors that target other RAS-mutant oncoproteins including zoldonrasib, a RAS (‘on-state’) G12D-selective inhibitor, and daraxonrasib, a pan-RAS (‘on-state’) inhibitor, have yielded promising activity.^124^ Daraxonrasib is compared with docetaxel in patients with previously treated *RAS*-mutant NSCLC (including mutations in codons G12, G13, and Q61 of *KRAS*, *HRAS*, or *NRAS*) in the phase III RASolve-301 clinical trial (NCT06881784).

*NRG1* fusion has emerged as a novel driver in NSCLC. In a registrational phase II study that included 12 different tumor types, 58% of which were NSCLC, zenocutuzumab achieved a 30% ORR.^125^

## Liquid biopsy and biomarkers

Concurrent tissue and ctDNA testing increases complete genotyping and shortens time to treatment initiation, supporting its role as a complementary diagnostic tool.^37^^,^^126^ The prognostic relevance of ctDNA detection and clearance is established,^127^ but sensitivity remains limited, especially with first-generation assays.^128^ Although more complex, tumor-informed methods offer higher sensitivity than tumor-agnostic methods.^129^ Efforts to improve its predictive value, including modeling MRD dynamics, are ongoing.^128^^,^^130^

The potential of ctDNA to guide or monitor therapy is under active study. In *EGFR*-mutated NSCLC, the APPLE trial confirmed the feasibility of ctDNA-based monitoring for emergent T790M mutations.^131^ Additional trials are assessing ctDNA-guided treatment (NCT04093167) and its correlation with imaging response (NCT05254782). The ECLIPSE tool identifies polyclonal metastatic dissemination, a feature of poor prognosis.^128^ Liquid biopsy technology is evolving beyond DNA analysis to incorporate methylation profiling, exosomes, and proteomics,^127^ though they cannot assess the tumor microenvironment or histologic transformation. Key challenges include defining optimal response metrics and predicting drug-tolerant persister clones.

While PD-L1 expression remains the main immunotherapy biomarker, it is imperfect.^37^^,^^57^ Tumor DNA analyses have highlighted the impact of co-mutations—such as *STK11* and *KEAP1* in *KRAS*-mutant NSCLC—which are associated with poorer responses to ICIs.^132^ Models with AI are being developed based on digital histopathology^133^ and radiomics.^134^ Additionally, nuclear imaging is exploring the use of novel tracers and radionuclides.^135^

### Small-cell lung cancer

Although 2-year survival for limited-stage SCLC (LS-SCLC) has improved, outcomes in extensive-stage (ES-SCLC) remain poor.^136^ The ADRIATIC trial established durvalumab consolidation after chemoradiotherapy as the new standard for LS-SCLC. In ES-SCLC, the first-line standard remains chemotherapy plus immunotherapy. The IMforte trial showed improved OS and PFS with lurbinectedin plus atezolizumab versus atezolizumab alone,^137^ and is now recommended.^138^ For post-platinum relapse, the bispecific T-cell engager tarlatamab [delta-like ligand 3 (DLL3) × CD3] improved OS in DeLLphi-304,^139^ joining lurbinectedin, topotecan, and amrubicin (Asia) as second-line options.^140^ Tarlatamab is also studied in the first-line setting (NCT06211036, NCT07005128) and in LS-SCLC (NCT06117774).

Other DLL3-targeted therapies include ADCs and next-generation engagers such as obrixtamig, gocatamig, QLS31904, and RO7616789. Gocatamig is also combined with ifinatamab deruxtecan in second-line trials (NCT06780137), while DLL3 CAR-T and radiotherapeutics are in early testing.^141^

B7-H3-targeted ADCs (e.g. ifinatamab deruxtecan, HS-20093, YL201) achieved ORR of 43%-64%,^142^, ^143^, ^144^, ^145^, ^146^ and TROP2 ADCs such as sacituzumab govitecan and tizetatug rezetecan reported ORRs of 42% and 33%, respectively.^147^^,^^148^ The bispecific ADC Izalontamab Brengitecan (EGFR/HER3) reached an ORR of 45%.^149^ Additional targets under study include seizure related 6 homolog, HER2/3, familial adenomatous polyposis, and mesothelin.^5^

Poly (ADP-ribose) polymerase inhibitors are also being explored (NCT04624204), and research is assessing whether they may benefit biomarker-selected populations, such as those with high Schlafen family member 11 expression, with sensitivity in preclinical models.^150^

VEGF inhibition is under study with ivonescimab plus chemotherapy, showing high ORR in phase Ib.^151^ BNT327, also targeting PD-L1 and VEGF, is also being studied (NCT06712355). Additional targets include lysine-specific histone demethylase 1 inhibitors,^152^ Proviral integrations of Moloney virus 2 inhibitors,^153^ and p53-targeted therapies (NCT04585750).

Despite progress, most patients derive limited benefit from immunotherapy.^154^, ^155^, ^156^ Recent research has underscored the role of epigenetic reprogramming,^157^ subtypes, and cell states in SCLC.^158^ However, this currently does not impact the treatment of SCLC.^159^

The use of prophylactic cranial irradiation (PCI) has declined with the increasing adoption of magnetic resonance imaging (MRI) surveillance. The ongoing phase III MAVERICK (NCT04155034) and PRIMALung (NCT04790253) trials are evaluating PCI versus MRI surveillance to determine the optimal approach.

Major challenges remain, including managing transformed SCLC and ensuring patients receive optimal first-line therapy, as rapid deterioration often precludes access to later-line agents.

## Collaborations and barriers

A key objective of the NY Lung Summit is to overcome barriers to clinical research and strengthen collaborations among academia, industry, and advocacy groups. Organizations such as the IASLC, ETOP-IBCSG Partners Foundation, and European Organization for Research and Treatment of Cancer exemplify this role, supporting investigator-initiated international trials, multidisciplinary care, and oncologist training.^160^^,^^161^ However, evolving European regulations have increased the complexity of trial initiation. Initiatives like COMBINE aim to harmonize and streamline processes.^162^^,^^163^

Financial constraints remain a major barrier to therapy adoption and clinical research participation. Linking reimbursement and quality metrics (e.g. Medicare Star ratings) to preventive care could incentivize early detection.^164^^,^^165^ Sustaining academic research capacity requires adequate funding for industry-sponsored trials and expanded use of community-based infrastructures, such as the National Cancer Institute (NCI) Community Oncology Research Program. Precompetitive, multi-sponsor funding models—pooled and product-agnostic—could support shared research platforms, biomarker development, and trial-enabling methods.

Pharma–academic partnerships remain essential for patient identification, while advocacy groups such as the EGFR Resisters and LUNGevity Foundation demonstrate the power of patient-driven real-world studies, though broader cross-national coordination is still needed.^166^^,^^167^ Addressing these structural and financial challenges through joint governance, transparent data sharing, and harmonized trial frameworks will be crucial to sustaining innovation and equity in global lung cancer research.

### Improving trial design

Panelists emphasized the need to simplify trial design, reduce administrative burdens, and ensure equitable patient access. Industry hesitancy toward head-to-head studies and the proliferation of ‘me-too’ trials dilute research capacity. Streamlined eligibility criteria, simplified specimen collection, and clearer regulatory guidance were identified as priorities. Strengthened investigator–sponsor communication, digital tools for patient-reported outcomes, and supportive interventions, such as tobacco-cessation programs, could improve both efficiency and participation. The FDA’s guidance on multiregional clinical trials aims to clarify standards for global applicability.^168^

Integrating biomarkers into enrollment is critical but constrained by the EU *In Vitro* Diagnostic Regulation, which mandates validation within controlled trials, slowing translational progress.^169^ Standardizing assays and expanding point-of-care sequencing could accelerate biomarker-driven research. Establishing a multi-stakeholder consortium, potentially United States National Institutes of Health (NIH)-supported, was proposed to harmonize standards and promote data sharing.

Accelerating trial initiation and recruitment in regions with robust infrastructure and expanding participation in LMICs were seen as essential for global relevance. Projects such as the FDA’s Orbis initiative may enhance international collaboration.^170^ Well-resourced sites, pragmatic trial designs, and public–private partnerships can support decentralization and inclusion.

Panelists also noted the need to adapt to regulatory reforms, improve data sharing, and implement adaptive or master protocols. Broader use of real-world data and stronger investigator-initiated networks will drive efficiency, inclusivity, and innovation in the next generation of clinical research.

### Disparities

Health care disparities—driven by structural, socioeconomic, and cultural inequities—remain a major barrier to equitable cancer outcomes.^171^ Around 70% of cancer deaths occur in LMICs, where deficits persist across prevention, diagnosis, and treatment.^172^, ^173^, ^174^ Addressing these gaps requires political commitment, provider education, public awareness, and patient-centered care models supported by targeted policy interventions.^171^

European initiatives such as the Organisation of European Cancer Institutes network, the Cancer Inequalities Registry, and European Society for Medical Oncology Vision 2025 are mapping disparities and promoting action.^175^, ^176^, ^177^ Despite the recent withdrawal of the FDA diversity guidance, maintaining diversity in clinical research remains a global imperative. Leveraging innovations from LMICs and ensuring that control arms reflect best available care are essential for ethical, generalizable results.

Improving trial access and infrastructure requires regional partnerships, sequencing hubs, and public–private collaborations to support logistics and data sharing. Engaging local governments and investigators early fosters sustainability and cultural alignment, mitigating risks of ethical imbalance or ‘research neocolonialism’.^178^

Strategic planning and continuous dialogue are vital to sustain progress. Forums such as the IASLC Pan-Africa Conference on Lung Cancer illustrate how empowering regional experts strengthens global collaboration and workforce development.^179^ Investing in young investigators and regional leadership will be key to building a more inclusive and resilient future in lung cancer research.

## Conclusion

Significant progress has been made in understanding and treating lung cancer, yet substantial challenges remain. Enhanced global collaboration is essential to delivering optimal care, supported by efficient research and inclusive, streamlined clinical trial designs. Sustained investment in research—including robust support for national and international agencies such as the NIH and NCI, the WHO’s International Agency for Research on Cancer, and the European Commission’s Horizon Europe program—is critical to driving innovation and improving patient outcomes. Meetings like the NYLCF Summit provide invaluable platforms for stakeholder engagement, fostering the partnerships and strategic planning needed to sustain momentum in the global fight against lung cancer.

## Panelists

Akimov M., Alatorre J., Altorki N., Anagnostou V., Arrieta O., Ballinger M., Bar J., Barlesi F., Bara I., Bazhenova L., Berg C., Bradley J., Borghaei H., Bunn P., Cantrell M., Calvet C., Cappuzzo F., Carbone D., Carter C., Corrales L., Dacic S., Daily J. Freeman, Diehn M., Doroshow D., Dziadziuszko R., Elghazaly H., Evans R., Felip E., Ferriari C., Flores R., Forde P., Fredrickson D., Gainor J., Gerber D., Govindan R., Gray J., Henschke C., Henick B., Herbst R., Heymach J., Hirsch F., Horn L., James L., Janne P., Kelly K., Kim Caius, Khorshid O., Kris M., Langer C., Leighl N., Levy B., Liu M., Lowy I., Mathias C., Marron T., Mitsodomi T., Mulshine J., Naidoo J., Norris K., Osarogiagbon R., Pass H., Passaro Antonio, Patel S., Paz-Ares L., Peled N., Peters S., Petruzelka L., Planchard D., Politi K., Popat S., Powell C., Pyenson B., Reckamp K., Ren H., Riely G., Rizvi N., Rohs N., Rolfo C., Rudin C., Rosenzweig K., Roy Upal B., Salvati M., Santhanagopal A., Sabari J., Shilo Shani, Shield M., Shum E., Singh H., Skoulidis F., Solomon B., Stahel R., Stiles J. B., Taioli E., Tenhunen Olli, Vokes E., Vokes N., Velcheti V., Wakelee H., Waqar S., Wistuba I., Winn R., Wolf Andrea, Wong K-K., Wozniak A., Wu Y-L., Yang J., Yang P-C., Yankelevitz D., Yoon B., Zulueta J.

## Declaration of generative AI and AI-assisted technologies in the manuscript preparation process

During the preparation of this work, the author used ChatGPT (GPT-5, OpenAI) to assist in language refinement, including correction of spelling and grammar. After using this tool, the author carefully reviewed and edited the text to ensure accuracy, originality, and alignment with the intended scientific meaning, and takes full responsibility for the content of the final manuscript.
